# Radiation Recall Supraglottitis Triggered by Pembrolizumab in a Patient With Metastatic Non-small Cell Lung Cancer: A Case Report

**DOI:** 10.7759/cureus.103073

**Published:** 2026-02-06

**Authors:** Brian Oh, Kunal Trehan, Shawgi Sukumaran

**Affiliations:** 1 College of Medicine and Public Health, Flinders Health and Medical Research Institute, Flinders University, Adelaide, AUS; 2 Department of Medicine and Cardiac and Critical Care, Flinders Medical Centre, Southern Adelaide Local Health Network, Adelaide, AUS; 3 Department of General Medicine, Royal Adelaide Hospital, Central Adelaide Local Health Network, Adelaide, AUS; 4 Department of Medical Oncology, Flinders Medical Centre, Southern Adelaide Local Health Network, Adelaide, AUS

**Keywords:** immune checkpoint inhibitors, immune-related adverse events, immunotherapy, pembrolizumab, radiation recall, supraglottitis

## Abstract

Supraglottitis is a serious, life-threatening condition that can present as an immune-related adverse event (irAE) in patients receiving cancer treatment with immune checkpoint inhibitors. Patients who have a history of receiving radiotherapy treatment to the head and neck region may have increased susceptibility of developing irAEs, a phenomenon known as radiation recall. We report a case of radiation recall supraglottitis in a patient receiving maintenance pemetrexed and pembrolizumab for the treatment of metastatic lung adenocarcinoma. He also had a previous history of right tonsillar squamous cell carcinoma for which he underwent adjuvant radiotherapy to the bilateral neck region. The patient presented with a three-week history of progressive fatigue, dysphonia, dysphagia, and sore throat, for which a flexible nasal endoscopy revealed significant swelling of the supraglottic structures. He was commenced on intravenous antibiotics and corticosteroids and was subsequently discharged after clinical improvement. However, the patient represented with persistent swelling of the supraglottis which later progressed to an acute airway obstruction requiring intubation. Despite being treated with high-dose intravenous steroids and interleukin-6 inhibitors, there was minimal clinical response. This report highlights the importance of recognising supraglottitis as a possible irAE in a patient who has had previous radiation treatment to the head and neck region.

## Introduction

In recent years, the widespread utilisation of immune checkpoint inhibitors (ICIs) has revolutionised the treatment of various malignancies, offering improved survival outcomes when utilised as monotherapy or in conjunction with other treatment modalities such as chemotherapy or radiotherapy [1]. Programmed death receptor-1 (PD-1) inhibitors such as pembrolizumab are amongst the most commonly used ICIs. However, immunotherapy-associated toxicities or immune-related adverse events (irAEs) have also become increasingly common, which can affect any organ or tissue [1,2]. Furthermore, previously irradiated tissues may be subject to increased risk of developing a delayed inflammatory response after being exposed to systemic drugs like ICIs, a phenomenon known as radiation recall [3]. Often, radiation therapy is utilised in the treatment of oropharyngeal cancers, which is a field close to critical upper airway structures. Thus, inflammatory responses in these regions may lead to clinically significant events such as critical airway compromise.

Although there are case reports of immunotherapy-related supraglottitis in the literature, supraglottitis as a manifestation of radiation recall secondary to systemic drugs has been rarely documented. There have only been two reported cases of radiation recall supraglottitis described by Wallenborn and Postma in 1984 [4] and Wiatrak and Myer in 1991 [5]. Here, we report a case of persistent supraglottitis with associated acute airway obstruction in a patient being treated with pembrolizumab for non-small cell lung cancer, with a prior history of radiotherapy for head and neck cancer.

## Case presentation

**Figure 1:**
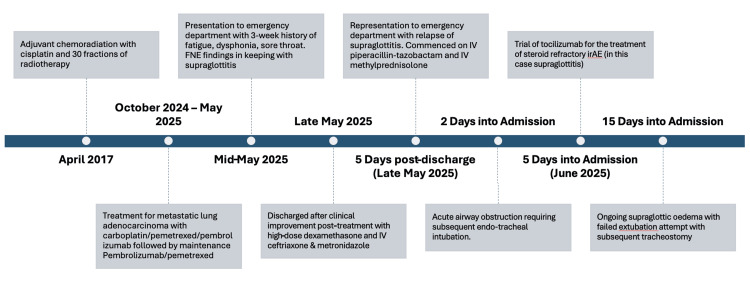
A timeline summarising the timing of chemoradiotherapy, initiation of immunotherapy for lung adenocarcinoma, symptom onset, response to steroids, relapse of symptoms, and airway compromise FNE: flexible nasal endoscopy; irAEs: immune-related adverse events

A 64-year-old man presented to the emergency department with a three-week history of progressive fatigue, dysphonia, dysphagia, and sore throat. He was undergoing treatment for metastatic lung adenocarcinoma with four cycles of carboplatin, pemetrexed, and pembrolizumab followed by maintenance pembrolizumab and pemetrexed. He also had a history of previous right tonsillar squamous cell carcinoma for which he underwent a right lateral oropharyngectomy and nodal clearance, followed by concurrent chemoradiation with adjuvant cisplatin and 30 fractions of radiotherapy (63 Grays) to the bilateral neck region in 2017 (Figure 1). On examination, he maintained normal oxygen saturations on room air with no work of breathing; however, on auscultation, there was reduced air entry with coarse crepitations in the left mid to lower zones with associated bronchial breathing. He was admitted and treated for community-acquired pneumonia with intravenous benzylpenicillin and oral azithromycin. 

**Figure 2:**
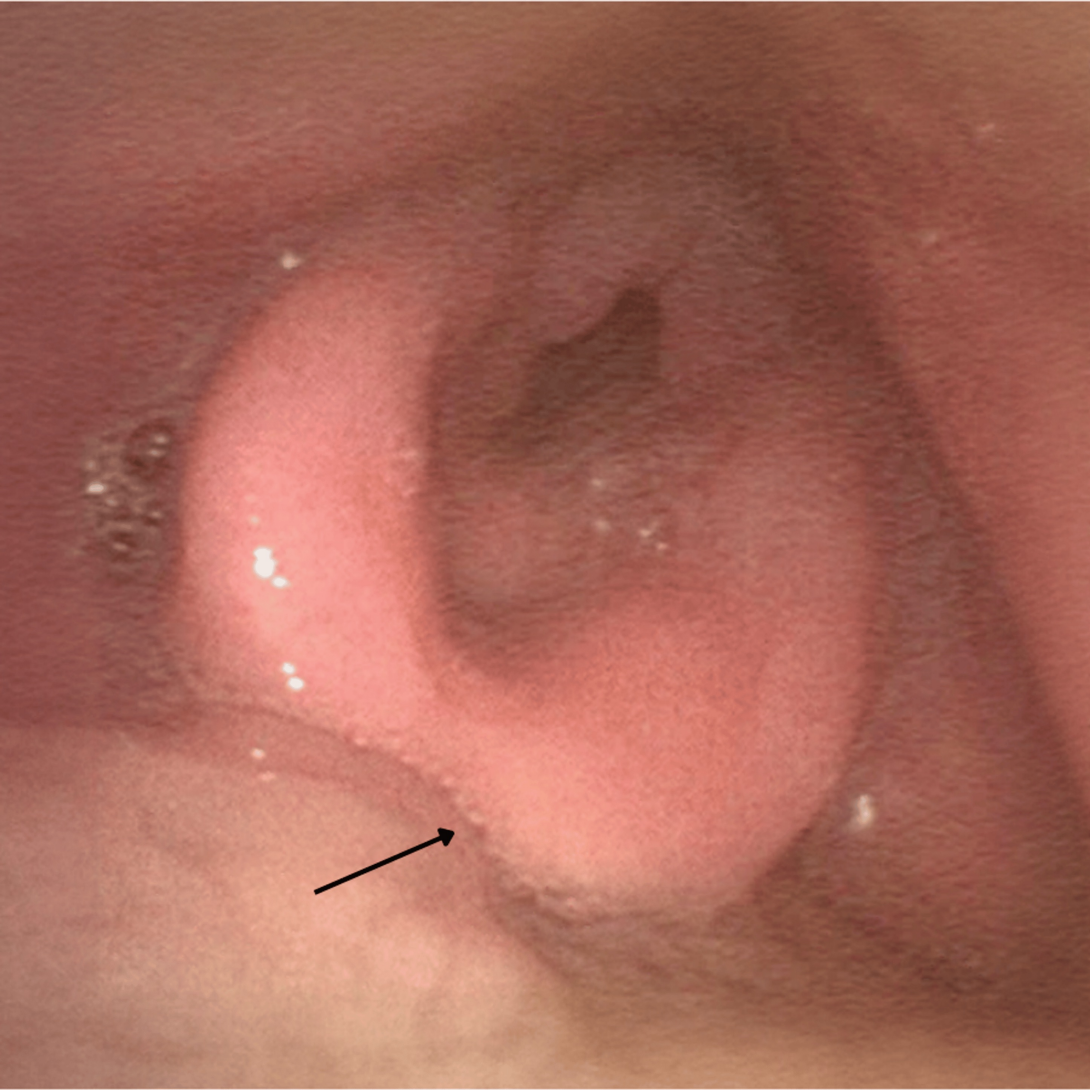
An FNE demonstrating severe supraglottitis at the time of initial presentation, with findings of significant epiglottic swelling and minimal visualisation of the true vocal cords, with significant pooling of secretions over the glottis region FNE: flexible nasal endoscopy

Further investigations included a flexible nasal endoscopy (FNE) by the Ear, Nose, and Throat (ENT) surgical team which demonstrated significant pooling of secretions over the glottis region and associated epiglottic swelling with minimal visualisation of the true vocal cords (Figure 2). There was no pus or collections visualised. The respiratory pathogen nucleic acid test (NAT) swab was negative for viral pathogens; however, a formal throat swab or sputum culture for bacterial microscopy, culture, and sensitivity was not conducted at this point in time. A clinical diagnosis of subacute supraglottitis was made although an infective aetiology was not excluded. The patient was commenced on high-dose dexamethasone and broad-spectrum antibiotics including intravenous ceftriaxone and metronidazole. Due to the history of dysphagia and high risk for aspiration, the patient was placed nil by mouth, and a nasogastric tube was inserted to commence enteral feeding for nutritional support. On serial flexible nasal endoscopies, there was a reduction in supraglottic swelling with progressive improvement of symptoms with medical management. After a successful taper and the cessation of steroids, the patient was later discharged with ongoing enteral feeding and oral antibiotics, with plans for outpatient clinic follow-up. Given the clinical presentation and supraglottic inflammation which responded to steroids, a clinical diagnosis of immunotherapy-induced supraglottitis was made. The localised nature of the inflammation was consistent with a clinical diagnosis of radiation recall.

**Figure 3:**
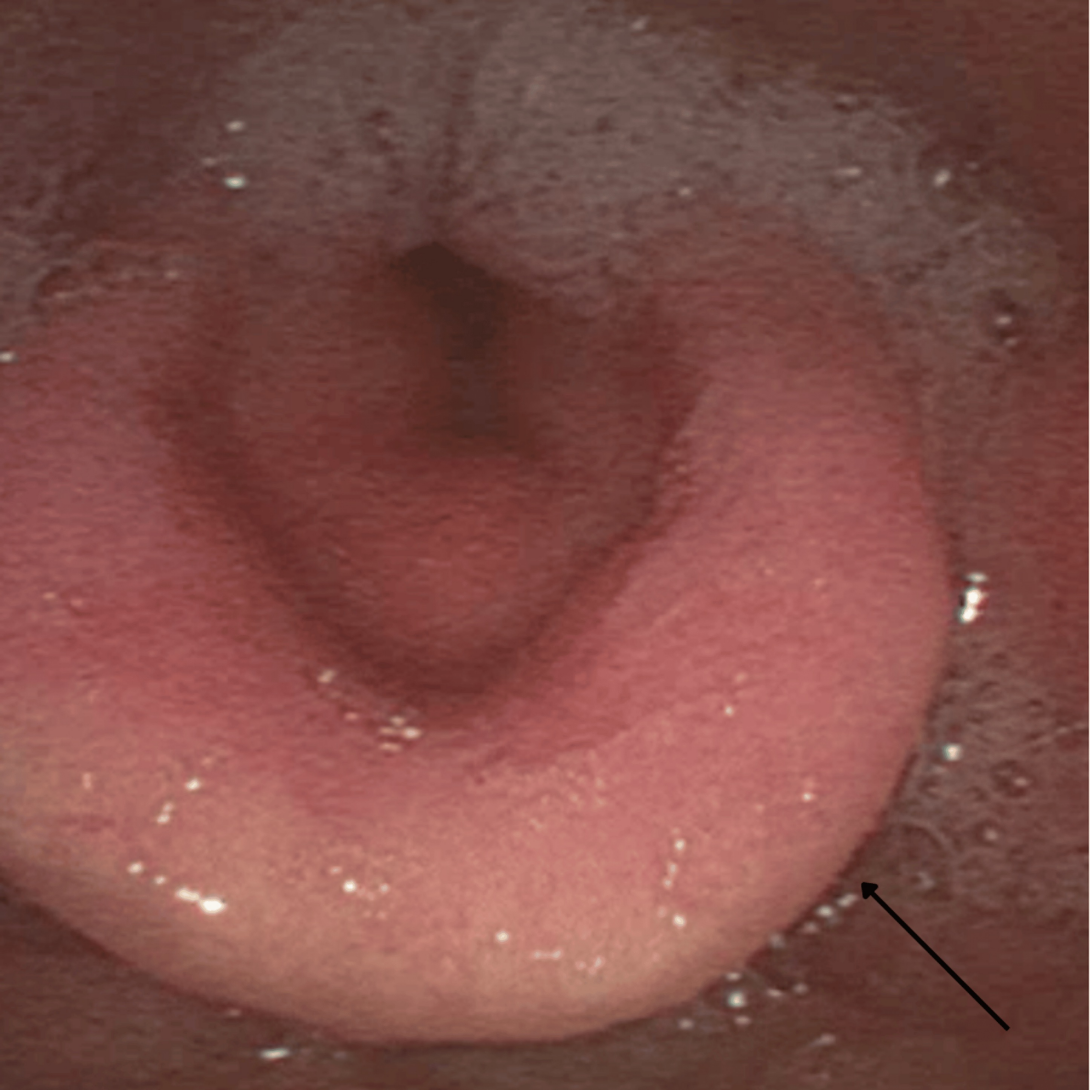
A repeat FNE demonstrating ongoing pooling of secretions with swollen epiglottis similar to the findings from his initial presentation FNE: flexible nasal endoscopy

Five days post-discharge, the patient re-presented to the emergency department with worsening dyspnoea and productive cough. On examination, he was saturating at 95% on 4L of oxygen with mild tachypnoea but without any respiratory distress. On auscultation, there was diffuse bronchial breathing with scattered bilateral lower lobe crepitations. Sputum culture was positive for oral flora without any identified bacterial pathogens, and the respiratory pathogen NAT swab was negative for viral pathogens. C-reactive protein was mildly raised at 69.6 mg/L; however, blood cultures were negative. A CT of the chest revealed progressive bronchial wall thickening and multi-lobar pneumonic changes with progressive interstitial oedema and pleural and pericardial effusions. N-terminal pro-B-type natriuretic peptide, a cardiac biomarker, was elevated at 856 ng/L with a transthoracic echocardiogram demonstrating a preserved ejection fraction of 55%. The cancer status was stable compared to previous staging scans. The patient was commenced on intravenous piperacillin-tazobactam for the treatment of pneumonia as well as diuresis for fluid overload. He was also commenced on high-dose steroids with intravenous methylprednisolone at 1 mg/kg to treat an irAE. A repeat FNE demonstrated ongoing pooling of secretions with recurrent swollen epiglottis similar to the findings from his prior admission (Figure 3).

Two days into his admission, a medical emergency response was activated for respiratory distress with stridor secondary to an acute airway obstruction. The patient was immediately transferred to the intensive care unit with subsequent bedside nasal endoscopy showing gross supraglottic oedema with a compromised supraglottic airway, requiring an urgent endotracheal intubation. However, no inflammation was identified past the supraglottic areas, and a subsequent bronchoscopy was largely unremarkable apart from a small amount of sputum in the left-sided airways. Given that corticosteroids and antibiotics alone were ineffective in controlling the supraglottic inflammation and oedema, as per departmental policy, a trial of an interleukin-6 inhibitor (tocilizumab) at 4 mg/kg was administered for the treatment of steroid-refractory irAE. Despite receiving two doses of tocilizumab, the patient had a minimal response and failed an extubation attempt. Subsequently, the patient underwent a tracheostomy to maintain his airway. However, he continued to deteriorate with progressive respiratory distress. A family meeting was held with the patient and his next of kin, with the consensus for transition to end-of-life care. He was subsequently taken off mechanical ventilation and later passed away. The cause of death was deemed as supraglottic oedema of multifactorial aetiology including irAE-related supraglottitis and respiratory tract compromise secondary to multi-lobar pneumonia, interstitial oedema, and pleural and pericardial effusions.

## Discussion

Over the years, the utilisation of immunotherapy drugs has demonstrated remarkable efficacy in the treatment of various advanced malignancies. Specifically, ICIs such as PD-1 inhibitors utilised alone or in combination with chemotherapy agents have shown improved survival outcomes in patients with advanced metastatic non-small cell lung cancer [1,2]. However, the modulation in the activity of T-cells with PD-1 blockade can lead to irAEs which can affect any organ or tissue in the body [1,2].

Supraglottitis is a serious condition characterised by the severe inflammation of the supraglottic structures of the larynx [6]. Adult supraglottitis may present with a slow onset of pharyngeal symptoms of a sore throat, dysphagia, odynophagia, and fewer severe respiratory symptoms [6]. In extreme cases, supraglottitis can cause an acute upper airway swelling and obstruction requiring prompt airway management. While an infective aetiology is the most common, non-bacterial causes secondary to malignancy, radiotherapy, and anti-cancer agents should be considered in such patients. As per current literature, only a few cases of supraglottitis have been reported as an irAE secondary to ICIs. Gascon et al. reported two cases of significant supraglottitis treated with PD-1 inhibitors (Table 1), in which the mild pharyngeal symptoms were incongruent with the diffuse severe supraglottic swelling and inflammation. Both patients were treated with high-dose systemic corticosteroids and intravenous antibiotics with serial FNEs showing improvement of supraglottic swelling with medical management, although the second patient had persistent supraglottic swelling without airway compromise due to ongoing immunotherapy treatment [2].

**Table 1:** Cases of ICI-induced supraglottitis The cases listed above are reported by Gascon et al. [2]. irAEs: immune-related adverse events; ICI: immune checkpoint inhibitors; PD-1: programmed death receptor-1; IgG4: immunoglobulin G4

	Primary histology	Treatment of malignancy (drug)	Effect mechanism	Complication or type of irAE	Treatment of irAE
Case 1	Stage IV metastatic melanoma	Nivolumab + relatlimab	PD-1 inhibitor + IgG4 monoclonal antibody	Supraglottitis and tracheitis, with relapse despite holding ICI therapy	High-dose systemic steroids and IV antibiotics
Case 2	Metastatic recurrent bladder cancer	Pembrolizumab	PD-1 inhibitor	Persistent supraglottitis	High-dose systemic steroids and IV antibiotics, followed by a slow oral steroid taper

An interesting aspect about this case is the history of previous tonsillar squamous cell carcinoma that was treated with adjuvant radiation to the bilateral head and neck region. We hypothesise that previously irradiated mucosal tissues may have had increased susceptibility to develop irAEs. This phenomenon in which systemic drugs cause a reactivation of latent radiation effects in previously irradiated tissues is known as radiation recall [3]. Although radiation recall reactions usually manifest as a cutaneous reaction such as dermatitis, inflammation of mucosal tissue in the supraglottic region has also been reported. In the current literature, only two previous cases have demonstrated radiation recall supraglottitis secondary to the administration of chemotherapy agents following radiotherapy to the neck region (Table 2) [4,5]. Furthermore, although radiation recall has been more commonly associated with cytotoxic chemotherapy agents, emerging data does suggest that ICIs have led to radiation recall reactions. A case series by Riviere et al. demonstrated three cases of radiation recall pneumonitis triggered by dual agent immunotherapy treatment, where all patients had very focal and asymmetric distribution of inflammation that was inconsistent with immunotherapy-induced pneumonitis [7]. Currently, the mechanism of a radiation recall response from ICIs is still poorly understood; however, the production of neo-antigens in irradiated tissue could theoretically result in increased inflammation through the heightened T-cell response against tumour antigens with PD-1 blockade [8].

**Table 2:** Reported cases of radiation recall supraglottitis The cases listed above are reported by Wallenborn and Postma [4] and Wiatrak and Myer [5].

Study	Patient demographics	Primary histology	Treatment of malignancy	Complication	Treatment of complication
Wallenborn and Postma [4]	47-year-old male patient	Stage IIA diffuse histolytic lymphoma	Initial: cyclophosphamide and Adriamycin, followed by radiation to both neck fields. On relapse: cytarabine and lomustine	Recurrent supraglottic oedema with associated cervical adenopathy and airway compromise	Intravenous dexamethasone (1 g every 6 hours)
Wiatrak and Myer [5]	4-year-old female patient	Pleomorphic rhabdomyosarcoma	Neoadjuvant dactinomycin, ifosfamide, and vincristine + adjuvant radiotherapy to the neck (60 Gy) followed by ifosfamide and vincristine	Epiglottic and supraglottic oedema with airway compromise	Tracheostomy followed by decannulation

An important aspect of this case is the discrepancy between the patient's symptoms and the initial FNE findings of supraglottic oedema with minimal visualisation of the true vocal cords. Given that there were only mild pharyngeal symptoms without stridor or other aerodigestive symptoms, this may suggest a baseline chronic supraglottic oedema for this patient before experiencing an acute flare in his representation. In addition, the minimal response to antibiotic therapy along with the negative upper airway viral and bacterial microbiology makes an infective aetiology less likely. Moreover, the initial responsiveness to systemic steroids along with the recurrence of his symptoms post-discharge and the cessation of steroids is also consistent with a clinically significant steroid-dependent, immune-mediated adverse event. In our case, the patient was treated with an interleukin-6 inhibitor (tocilizumab) as per departmental policy. However, we recognise that other agents that inhibit TNF-alfa (infliximab) could have been used in this setting as per the existing American Society of Clinical Oncology/European Society for Medical Oncology (ASCO/ESMO) guidelines [9,10]. Furthermore, the concurrent lower respiratory tract compromise secondary to multi-lobar pneumonia, interstitial oedema, and pleural effusions may have also contributed to his clinical decompensation, including the acute airway obstruction caused by worsening supraglottic swelling refractory to both steroids and interleukin-6 inhibitors.

Overall, this case highlights the importance of recognising supraglottitis as a potential manifestation of irAEs in a patient who has received radiation therapy to the head and neck area previously. Chemotherapeutic agents such as pemetrexed are known to be associated with radiation recall [11,12]; however, this is less likely in this case given the clinical presentation and initial significant response to corticosteroids. Although it can be challenging to definitively distinguish between a de novo immune reaction and an immune-mediated recall reaction, given that inflammation occurred in an area of previous irradiation, along with the incomplete and eventually refractory response to steroids, a radiation recall supraglottitis may have played a role in this patient's presentation. This case adds to the emerging entity of radiation recall reactions secondary to ICIs. Prompt diagnosis with ENT involvement, regular airway monitoring, and the early initiation of treatment along with the cessation of immunotherapy is vital in the management of this condition.

## Conclusions

Supraglottitis is a serious and potentially life-threatening diagnosis that can manifest as an irAE in patients undergoing systemic treatment with ICIs. Although radiation recall manifesting as supraglottitis is a rare clinical entity, it is a diagnosis that should be considered in patients with a past history of radiotherapy undergoing treatment with ICIs. A high index of suspicion and early intervention are important to minimise morbidity and mortality. Clinicians should take into account a patient's prior history of radiotherapy before considering the initiation of systemic treatment with immunotherapy.
